# Experimental Usutu virus infection in Eurasian blackbirds (*Turdus merula*)

**DOI:** 10.1038/s44298-025-00133-w

**Published:** 2025-06-20

**Authors:** Gianfilippo Agliani, Imke Visser, Eleanor M. Marshall, Giuseppe Giglia, Erwin de Bruin, Ruben Verstappen, Tjomme van Mastrigt, Felicity Chandler, Reina Sikkema, Henk van der Jeugd, Marion P. G. Koopmans, Andrea Gröne, Barry Rockx, Judith M. A. van den Brand

**Affiliations:** 1 Division of Pathology, Faculty of Veterinary Medicine, Utrecht University, Utrecht, the Netherlands; 2 Department of Viroscience, Erasmus Medical Center, Rotterdam, the Netherlands; 3 Department of Veterinary Medicine, University of Perugia, Perugia, Italy; 4 Department of Animal Ecology, Netherlands Institute of Ecology (NIOO-KNAW), Wageningen, the Netherlands; 5 Vogeltrekstation - Dutch Centre for Avian Migration and Demography (NIOO-KNAW), Wageningen, The Netherlands; 6 Wildlife Ecology and Conservation Group, Wageningen University & Research, Wageningen, the Netherlands; 7 Dutch Wildlife Health Centre (DWHC), Utrecht University, Utrecht, The Netherlands

**Keywords:** Microbiology, Pathogenesis

## Abstract

Usutu virus (USUV) is a zoonotic arbovirus causing disease in wild birds and humans. Eurasian blackbirds (*Turdus merula*) are highly susceptible to infection, developing severe lesions with high mortality rates during outbreaks. The pathogenesis and clinical course of the acute disease in birds are not known. Therefore, six blackbirds were infected with two USUV lineages (Africa 3 and Europe 3). The blackbirds were monitored for clinical signs of disease and activity levels and were sampled for virus detection. All infected blackbirds showed severe disease after 4 to 7 days, with no significant differences between the lineages. Reaching a humane endpoint, the blackbirds were euthanized, and tissue samples were collected for histopathology and virology. At histopathology, lesions were seen in the main target organs with the associated presence of infectious virus and viral antigen. In conclusion, blackbirds can be infected experimentally with USUV and show similar disease as seen in natural infection.

## Introduction

Usutu virus (USUV) is an emerging zoonotic arbovirus belonging to the family *Flaviviridae*, genus *Orthoflavivirus*. The enzootic transmission cycle is maintained in nature by the infection of mosquito vectors, mainly *Culex* spp., and of several avian species as hosts, although it remains to be determined which avian species are the main reservoir hosts. Since its first detection in Europe, in Austria in 2001^1^, USUV has been responsible for outbreaks of mortality in birds in several European countries^2–5^, leading to the decline of wild birds populations^6^. Despite the broad avian host range of USUV infection^7^, Eurasian blackbirds (*Turdus merula*) and great gray owls (*Strix nebulosa*) are particularly susceptible to disease, as evidenced by the development of severe pathological changes and epidemiological data, suggesting a higher mortality compared to other avian species^1,2^. The pathological changes associated with natural USUV infection in blackbirds have been previously investigated based on necropsies of birds found dead or severely ill^8,9^, representing the end stage of disease, but the pathogenesis and clinical progression of the disease are not fully understood. USUV infection can occasionally spill over from the enzootic bird-mosquito transmission cycle to other incidental hosts, including humans^10,11^. USUV infection in humans is most often asymptomatic, but can lead to severe neurological disease in immunocompromised individuals^11^. Considering the impact on wild bird populations and potential threat to human health, modeling USUV infection in the avian host becomes relevant to obtain information on the role of different avian species in the transmission cycle of the virus, that can translate in a more targeted active and passive surveillance and early detection in the context of future outbreaks of the disease.

Experimental infections with USUV have been performed in chickens (*Gallus gallus domesticus*)^12^, red-legged partridges (*Alectoris rufa*)^13^, and magpies (*Pica pica*)^14^. These animals developed no viremia or lesions, and only a limited antibody response was detected in chickens^15^, and therefore were deemed to be resistant to infection, although there is evidence for natural USUV infection in sentinel chickens^16^. Experimental USUV infection in domestic geese (*Anser anser*) resulted in the development of viremia and brain lesions but no mortality^17^. USUV infection of domestic canaries (*Serinus canaria*)^18,19^ and house sparrows (*Passer domesticus*)^20^ resulted in the development of viremia, but at the same time, with a low mortality rate, identifying house sparrows as a potential reservoir of the infection. However, experimental infection of the most affected species during natural outbreaks, such as blackbirds, has not been performed before.

The aim of this study is to investigate whether subcutaneous inoculation of USUV Africa 3 (Afr3) and Europe 3 (Eu3) can recapitulate the clinical disease in Eurasian blackbirds (*T. merula*) and the pathology and pathogenesis associated with natural USUV infection in a highly susceptible species. To do so, we infected six Eurasian blackbirds with USUV. To highlight potential differences in pathogenicity, we used two different USUV lineages, Afr3 and Eu3, that are respectively enzootic and periodically introduced in avian species in the Netherlands^21^. Following USUV inoculation, we monitored the progression of the disease, determined kinetics of viremia and viral shedding and performed a pathological investigation to determine tissue tropism after euthanasia or death. This data allows for a comparison of experimental USUV infection with natural USUV infection of blackbirds, to evaluate the clinical disease and to obtain insights into the pathogenetic mechanisms that underlie the development of fatal disease in this specific bird species.

## Results

### Experimental USUV infection caused severe, acute illness and mortality in blackbirds

**Fig. 1:**
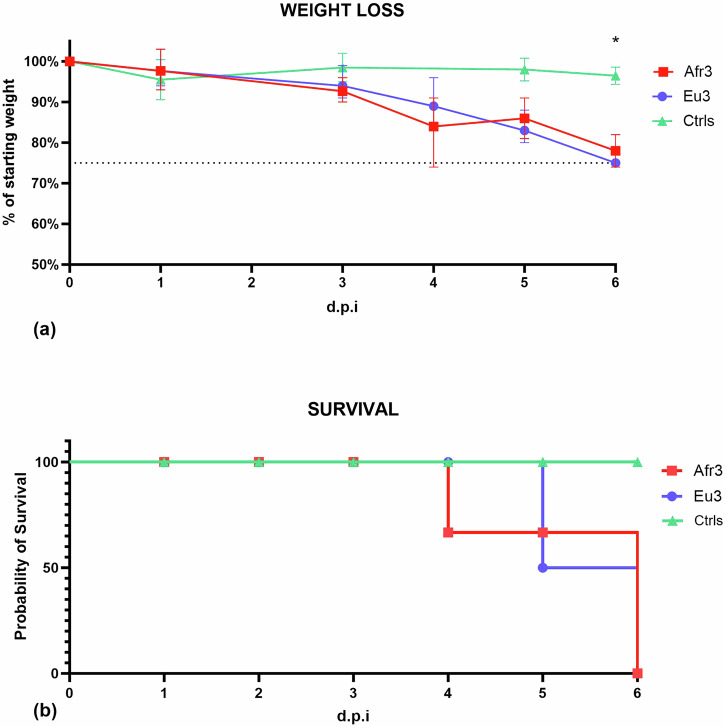
Weight loss and survival in blackbirds. **a** Weight loss is expressed in % of initial weight. The humane endpoint was set at 75% of initial weight (dashed line). Results averaged from three individuals infected with USUV Afr3 and Eu3 and two control individuals, bars represent SD. Statistically significant difference was observed between the Eu3 and ctrls group on 6 d.p.i. as indicated by the asterisk *p = 0.03. **b** Survival curves of blackbirds. No statistically significant difference was observed in survival among the three groups of blackbirds.

**Fig. 2:**
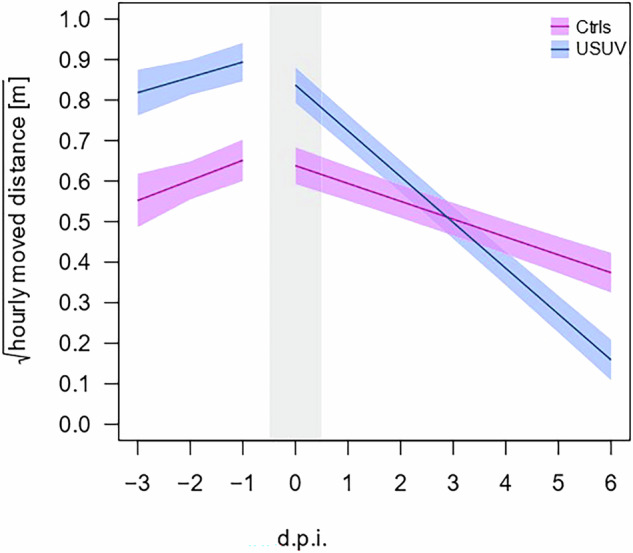
Levels of activity decrease more in USUV-infected blackbirds than in control birds. GAMM fit on the individual blackbirds’ square-root transformed hourly-total moved distances (in meters) for each day. Due to the small sample size, we combined the Afr3 and Eu3 infected birds in one treatment group (USUV-infected), and compared these birds to the control group. For visualization purposes, the time of day is fixed to the average value within the total data set (whilst the model corrects for diurnal activity patterns). Intervals around the lines depict standard errors of the predicted daily values. The gray rectangle indicates the day on which the experimental infection took place.

To investigate clinical disease progression induced by USUV infection, weight loss, development of clinical signs of infection, and levels of activity in the blackbirds were monitored. The average weight loss in the infected groups reached 75% of the starting weight within only 6 days, with only a statistically significant difference between Eu3 and the control group on 6 d.p.i. (*p* = 0.03) (Fig. 1a) on mixed effect analysis (alpha = 0.05). All infected blackbirds developed non-specific clinical signs of discomfort such as hunched posture, ruffled feathers and partially closed eyes between 3 and 6 d.p.i. The rapid progression of clinical signs leads to all blackbirds either reaching the humane endpoint or succumbing to the infection between 4 and 7 d.p.i. (Fig. 1b). No clinical signs of infection were observed in the control blackbirds. Hourly activity levels decreased following the experimental infection, but not prior to it, in USUV-infected blackbirds. While there was a reduction in activity in the mock-infected animals, likely due to the repeated sampling, this reduction was much less steep in mock-infected compared to USUV-infected blackbirds (GAMM: days post inoculation × stage of experiment × treatment: *F* = 8.85, df = 1, *P* = 0.0030; see supplementary materials; Fig. 2).

### Blackbirds potentially shed the virus through several routes

**Fig. 3:**
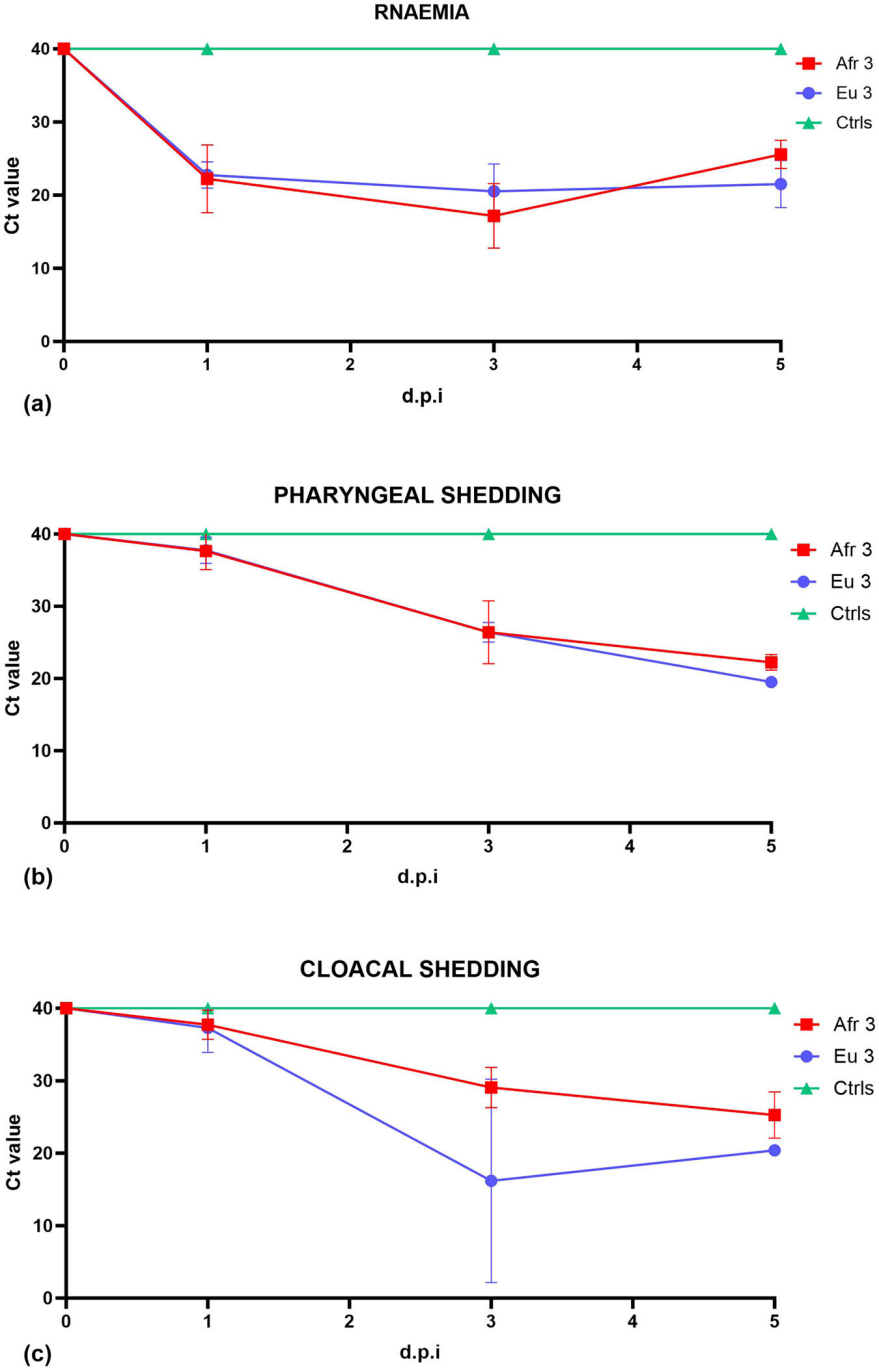
Blackbirds developed RNAemia and shed the USUV genome through the pharyngeal and cloacal route. Results averaged from three individuals infected with Afr3 and Eu3 viruses. **a** Levels of RNAemia expressed in Ct value. **b** Amount of virus shed through pharyngeal route expressed in Ct value, **c** Amount of virus shed through cloacal route expressed in Ct value. Error bars represent SD.

To investigate the patterns of USUV viremia and shedding in the blackbirds, we tested blood samples, pharyngeal and cloacal swabs and feathers collected before and after inoculation by RT-qPCR for the presence of viral genome. Viral genome was detected in the blood (RNAemia) of all infected blackbirds from 1 d.p.i., with average Ct values of 22.23 for Afr3-infected blackbirds and 22.77 in Eu3-infected blackbirds (Ct values in Supplementary Table 1). The average values of RNAemia peaked at day 3 in Afr3-infected blackbirds (17.16) as well as in Eu3-infected blackbirds (20.53) (Fig. 3a). We detected progressively increasing amounts of viral RNA in pharyngeal (Fig. 3b) and cloacal swabs (Fig. 3c), reaching Ct values of respectively 22.25 and 25.27 for Afr3-infected blackbirds and 19.51 and 20.41 for Eu3-infected blackbirds (Ct values Supplementary Tables 2, 3), suggesting virus shedding through the oral and cloacal route. No statistically significant difference was observed between the two USUV lineages for RNAemia (*p* = 0.3), pharyngeal (*p* = 0.07), and cloacal (*p* = 0.2) shedding, although, for all these parameters, there seems to be a trend towards a higher average amount of viral RNA in Afr3-infected blackbirds compared to Eu3-infected blackbirds. Relatively low levels of viral RNA (the lowest Ct value registered from BB4 on day 3 p.i. is 31.30) were detected in breast feathers. No viral RNA was detected in the breast feathers of control blackbirds.

### USUV replicates in several organs and tissues

**Fig. 4:**
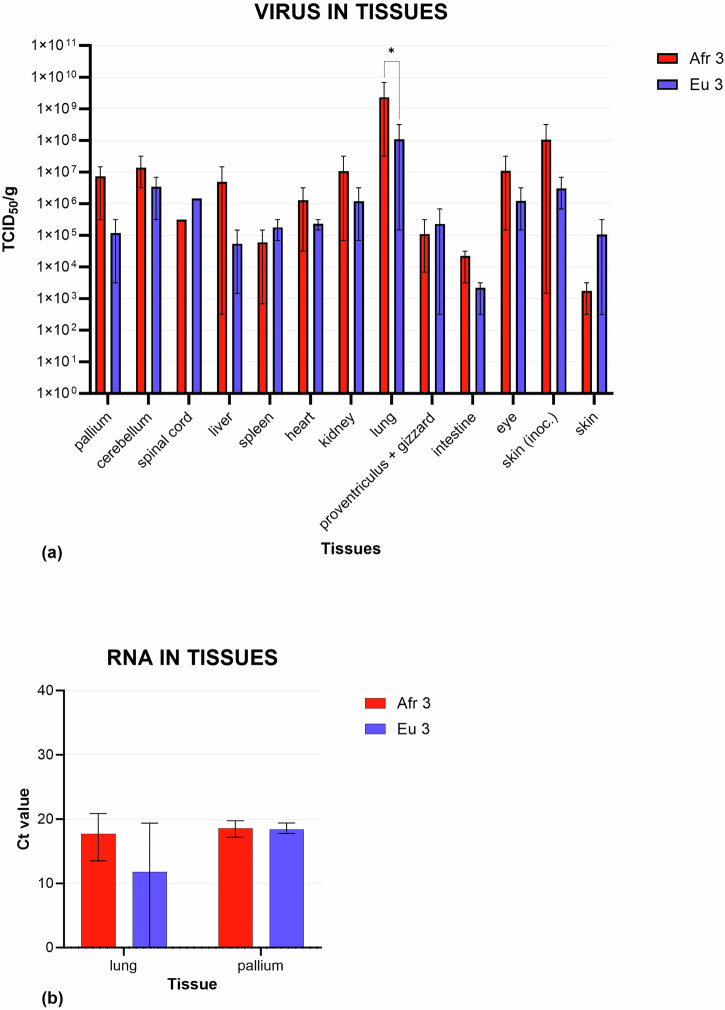
Infectious virus and viral RNA detected in tissues. **a** The amount of infectious virus in several tissues collected postmortem from the infected blackbirds is expressed in TCID_50_/g of tissue. Results averaged from three individuals for USUV Afr3 and USUV Eu3 groups, bars represent SD. A statistically significant difference (*p = 0.02) was observed between USUV Afr3 titer (2,3 × 10^9^ TCID_50_/g) and USUV Eu3 titer (1 × 10^8^ TCID_50_/g). **b** The amount of viral RNA detected in the lung and pallium of infected blackbirds is expressed in Ct value. Results averaged from three individuals for Afr3 and Eu3 groups, bars represent SD. No statistically significant differences were observed in either of the two tissues (lung *p* = 0.1; pallium *p* = 0.8).

To determine the tropism and distribution of USUV in the blackbirds, we tested tissues from a wide range of organs, and found infectious virus for both USUV Afr3 and USUV Eu3 lineages in many of them (Fig. 4a). No significant differences between USUV Afr3 and USUV Eu3 titers were detected in any organ except for the lung (*p* = 0.02), in which USUV Afr3 grew to higher titers compared to Eu3 (Supplementary Table 5). Looking at various organs, the central nervous system (CNS) had high titers of USUV Afr3 in both pallium and cerebellum. Within visceral organs, titers in the liver varied greatly within the USUV Afr3 group, while this variation was less prominent in the USUV Eu3 group. The inoculation site showed detectable titers for all animals in both groups, but only one animal per group had detectable USUV titers in skin sampled from a distant site, which was higher for USUV Eu3 compared with USUV Afr3.

To compare with procedures carried out during passive surveillance on natural infection in the Netherlands, we investigated the presence of USUV RNA in lungs and pallium through an RT-qPCR assay. Viral RNA was detected in the lung and pallium, in accordance with the detection of replicating virus by tissue titration. Higher viral RNA loads were detected in the lung for USUV Afr3-infected blackbirds compared to Eu3-infected blackbirds (Supplementary Table 6), but this difference was not statistically significant (*p* = 0.1). In the pallium, a higher amount of viral RNA was detected in Afr3-infected blackbirds compared to Eu3-infected blackbirds, but the difference was not statistically significant (*p* = 0.8) (Fig. 4b).

**Table 1:** Severity of USUV-associated lesions in the main target organs

	Afr3			Eu3		
	BB1	BB2	BB4	BB3	BB5	BB6
**Encephalitis**	1	1	1	0	0	0
**Neuronal necrosis**	2	2	2	1	1	1
**Hepatitis**	1	0	2	1	0	2
**Liver necrosis**	1	2	2	1	0	1
**Splenitis**	1	2	2	1	1	1
**Spleen necrosis**	2	3	3	2	2	1
**Myocarditis**	0	0	0	0	0	0
**Heart necrosis**	0	0	0	0	0	0
**Pneumonia**	1	1	1	1	1	0

Histopathological lesions in main target organs were graded as follows, 0 = normal tissue; 1 = mild (<20% of tissue affected); 2 = moderate (20–60% of tissue affected); 3 = severe (>60% of tissue affected), in all blackbirds (BB) infected with Afr3 (BB1, BB2, and BB4), Eu3 (BB3, BB5, and BB6).

### USUV induces macroscopical and histological lesions in target organs

**Fig. 5:**
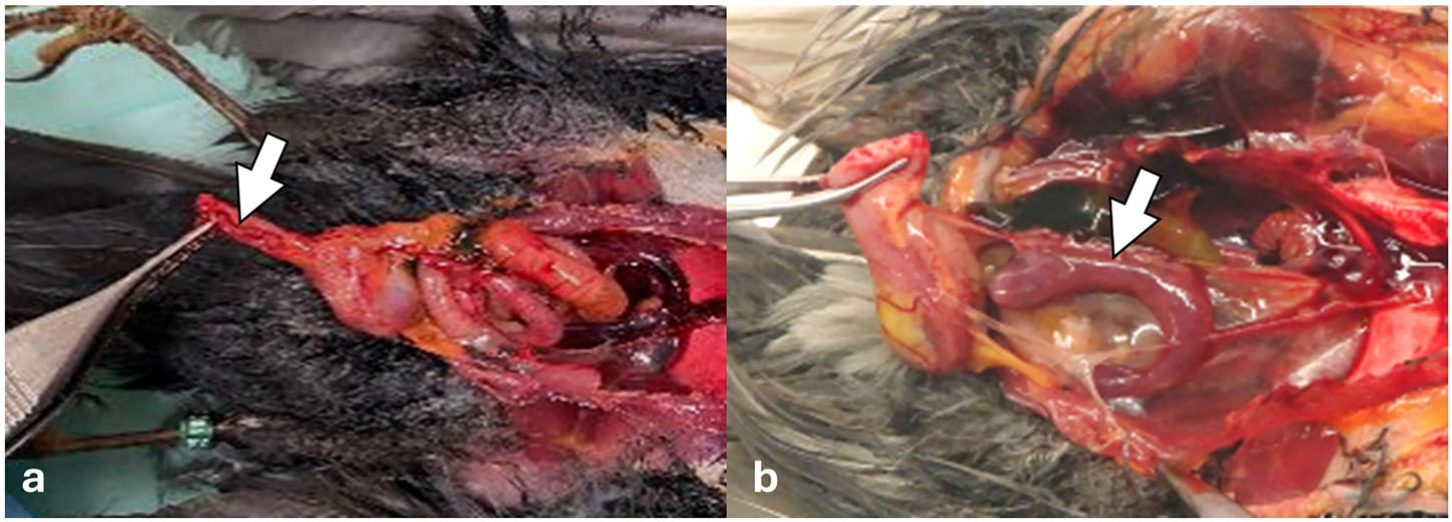
USUV-associated macroscopical lesions in representative control and infected blackbirds. Comparison of **a** a normal-sized spleen (arrowhead) in a control blackbird compared to **b** an enlarged spleen (arrowhead) in an infected blackbird.

All infected blackbirds showed variable degrees of splenomegaly, as was noticed during necropsy (representative image: Fig. 5). Histologically, the spleen of all blackbirds showed splenic necrosis associated with mild to moderate lymphoplasmacytic splenitis, with higher grades in USUV Afr3 3-infected blackbirds (Table 1). In all infected blackbirds, the liver showed variable grades of hepatocellular degeneration and necrosis, with higher grades in Afr3-infected blackbirds. In four blackbirds, belonging to both USUV lineages, hepatic necrosis was associated with mild periportal lymphoplasmacytic hepatitis, with similar severity scores for both USUV Afr3 and USUV Eu3. In the brain, foci of acidophilic neuronal necrosis associated with gliosis was observed in the pallium of all blackbirds, with a higher grade in Afr3-infected compared to Eu3-infected blackbirds. A mild encephalitis characterized by thin lymphoplasmacytic perivascular cuffs was observed in Afr3-, but not in Eu3-infected blackbirds. The heart did not show any characteristic lesions referable to USUV infection in any of the infected blackbirds, apart from a mild degeneration of the cardiomyocytes. A mild lymphoplasmacytic interstitial pneumonia was observed in all blackbirds except one.

### USUV exhibits tropism for several tissue and cell types

**Fig. 6:**
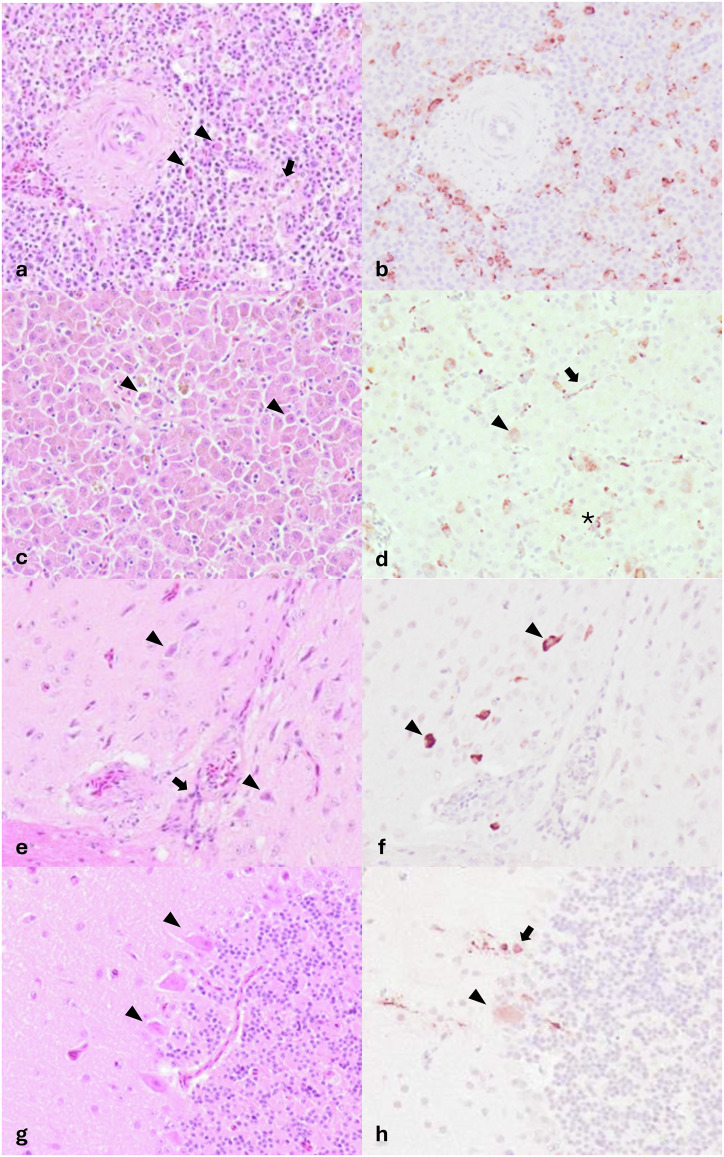
USUV-associated microscopical lesions and cell tropism. USUV-associated lesions (H&E) (**a**, **c**, **e**, **g**) and USUV tropism (**b**, **d**, **f**, **h**) in the main target organs of USUV Afr3-infected blackbirds. **a**, **b** Spleen of blackbird n.4: **a** small multifocal areas of necrosis in the spleen (arrows) and infiltration of plasma cells and mott cells (arrowheads). **b** USUV antigen in the cytoplasm of large mononucleated cells morphologically compatible with splenic macrophages (**c**, **d**) Liver of blackbird n.4: **c** diffuse vacuolization and pigment deposition in the cytoplasm of hepatocytes, multiple cells with pyknotic nuclei and hyper eosinophilic cytoplasm (single cell necrosis) (arrowheads). **d** USUV antigen in the cytoplasm of hepatocytes (arrowhead), endothelial lining of the sinusoids (arrow) and Kupffer’s cells (asterisks). **e**, **f** Brain, pallium of blackbird n.1: **e** multiple neurons undergoing acidophilic neuronal necrosis (arrowheads) and mild perivascular infiltration of mononucleated leukocytes (arrow). **f** USUV antigen in the cytoplasm of neurons (arrowheads). **g**, **h** Brain, cerebellum of blackbird n.1: **g** Multiple Purkinje cells in variable degree of degeneration from chromatolysis to acidophilic neuronal necrosis (arrowheads). **h** USUV antigen in the cytoplasm of Purkinje cells (arrowhead) and adjacent neurons of the molecular layer (arrow).

The presence of USUV antigen was observed throughout the tissues of all USUV-infected blackbirds. The score reflecting the amount of USUV antigen (number of positive cells and their distribution) in each tissue is reported in Table 2 and appears overall higher in Afr3-infected blackbirds compared to Eu3-infected blackbirds. In all examined tissues, endothelial cells lining vessels were multifocally positive and a diffuse positivity of mononucleated cells morphologically compatible with circulating monocytes was observed within the vessel lumina (Fig. 6). In the liver, virus antigen (when present) was located in the hepatocytes, Kupffer’s cells, sinusoidal endothelial cells, and spindle cells in arterial walls (Fig. 6f). In the spleen, virus antigen was observed in the cytoplasm of large cells morphologically compatible with cells of the mononuclear-macrophage line, as well as in spindle cells in the wall of small arterioles (Fig. 6h). In the brain, virus was present in the neurons and glial cells of the pallium and brainstem and in Purkinje cells of the cerebellum (Fig. 6b). Positive neurons were also observed in the spinal cord. Few infiltrating mononuclear cells showed cytoplasmic positivity in the heart. In the kidney, the antigen was observed in the cytoplasm of multiple endothelial cells in the glomeruli, as well as tubular epithelial cells and interstitial spindle cells. In the lung, alveolar macrophages and mononucleated infiltrating cells were positive for USUV antigen. Along all parts of the gastrointestinal tract, USUV antigen was present in the cytoplasm of epithelial cells in the mucosa as well as in spindle cells of the submucosal layer and in neurons of the myenteric plexus. In the skin of four blackbirds, the dermis and the pulp and shafts of the feather follicles showed positive cells.

**Table 2:** Amount of USUV antigen in tissues

	Afr3			Eu3		
	BB1	BB2	BB4	BB3	BB5	BB6
**Pallium**	3	3	1	2	1	3
**Cerebellum**	3	3	1	2	1	2
**Spinal cord**	2	1	1	2	2	3
**Ischiatic nerve**	0	0	n.a.	0	0	0
**Liver**	0	0	3	1	1	0
**Spleen**	1	0	3	1	0	1
**Heart**	0	1	2	1	2	2
**Kidney**	2	0	3	2	1	2
**Lung**	1	1	3	2	2	2
**Large Int**	3	3	3	3	2	2
**Small Int**	3	n.a.	3	3	2	2
**Proventriculus and gizzard**	2	2	3	n.a.	1	2
**Eye**	n.a.	1	1	n.a.	1	1
**Skin**	0	0	3	2	1	1

The amount of USUV antigen was assessed on IHC with a two-tier scoring system as follows, grade 0 = absence of antigen; grade 1 = low amount of antigen; grade 2 = moderate amount of antigen; grade 3 = high amount of antigen (n.a. not available), in all infected blackbirds, infected with USUV Afr3 (BB1, BB2, and BB4), USUV Eu3 (BB3, BB5, and BB6).

## Discussion

The present study aimed to investigate whether subcutaneous inoculation of USUV Afr3 and Eu3 can recapitulate clinical disease and associated histopathological changes in Eurasian blackbirds (*T. merula*). To obtain novel insights into the dynamics and pathogenesis of USUV infection in blackbirds, we compare clinical and pathological data from this study to what is currently known about natural USUV infection in blackbirds. However, from the investigation on natural infection, very little is known about the clinical progression of the disease, mostly because all the available data comes from surveillance studies based on postmortem evaluation of wild blackbirds collected after death. The experimentally infected blackbirds in the present study rapidly developed severe signs of disease (ruffled feathers, partially closed eyes and hunched posture), weight loss and marked reduction of activity shortly after the infection, resulting in lethal disease within a few days. Such clinical signs of disease are seldom described in the course of natural infection, only in those few occasions when diseased wild blackbirds were observed when still alive^3,9^. In naturally infected blackbirds, neurological symptoms such as opisthotonos, seizures and paralysis are also described^3,9,22^. However, in our study, we did not observe overt signs of neurological disease. This discrepancy with natural infection may be due to the rapid onset of disease and acute death of the blackbirds. Greater distribution of antigen and histopathological changes, as well as a higher virus titers in the liver, and not in the CNS, observed in the early stage of the infection, suggest that the virus might spread to the visceral organs first causing severe lesions that could lead to succumb to the infection at this early stage, before the virus can cause severe damage to the CNS.

USUV is known to spread to many different tissues where it targets different cell types and causes lesions mainly represented by necrosis and lymphoplasmacytic inflammation^1,9^. In the present experiment, the tissue and cell tropism is recapitulating what was observed in the natural infection and, for the first time, tropism for neurons in the spinal cord is reported. The acute death of the blackbirds in the present experiment also potentially reflects on the virus tropism and nature of the observed histopathological changes. The histopathological changes in this study were mainly represented by severe necrosis with a very mild inflammatory component, the very rapid progression of the infection did not leave enough time to recruit lymphocytes and plasma cells to the infected tissues that usually characterize inflammation during natural infection^9^. This also suggest that naturally infected blackbirds might survive the infection longer, introducing the need to investigate differences in the dose of virus inoculated as well as the immunological status of the blackbirds that can be influenced by the genetic background, especially for captive bred blackbirds as the ones in the present study, or by stress associated with housing in a BSL3 laboratory.

On average, visceral organs showed lower amounts of virus compared to the CNS, confirming the CNS as one of the main targets of USUV, as observed during natural infection^8,9^. The only exception is represented by a high load of virus in the lung, also confirmed in RT-qPCR assay. However, comparing IHC investigations previously performed in naturally infected blackbirds^9^, the parenchymal cells of the lungs usually have very little or no viral antigen, a finding that is also confirmed in the present experiment, where the majority of positive cells are represented by mononucleated circulating cells in the blood vessels. Therefore, the high viral load in the lung in the absence of virus antigen in the cytoplasm of parenchymal cells could be explained by the high levels of viremia and by the fact that the lung is a highly vascularized organ. This highlights the importance of combining virological and histopathological investigations to study the pathogenesis and tissue tropism of USUV in blackbirds.

In the present study, blackbirds were infected with two different USUV lineages currently circulating in the Netherlands among wild birds. No statistically significant differences emerged from the comparison of the two lineages in any of our analyses, except for the virus titer in the lung. The lack of detectable significant differences could be due to the small group size of this experiment, imposed by the limited commercial availability of blackbirds. Comparison of lineages in natural infection shows that there are no significant differences in the severity of histopathological changes between the Afr3 and Eu3^9^. Our data suggested that the Afr3 lineage may replicate more efficiently than the Eur3 lineage in blackbirds, although we cannot rule out that this was a result of a different inoculation dose.

USUV is a vector-borne pathogen transmitted by infected *Culex* spp. mosquitoes during a blood meal, therefore, the dynamics of the viremia in the infected host represent relevant information for better understanding the role of blackbirds in the transmission cycle of the virus. In the present experiment, both viral lineages induced high levels of viremia in the blackbirds, already detectable at 1 d.p.i. and peaking at 3 d.p.i. The high levels of viremia indicate blackbirds’ suitability in the transmission of the virus to the vector; however, the short timeframe of the viremia and the acute progression of the disease to death might limit the chances of a competent vector getting in contact with blackbirds. As discussed earlier, the experimental setting (dose, route and site of the virus injection) does not completely reflect natural transmission, impacting the patterns and dynamics of the viremia and subsequently influencing the role of the blackbirds in the transmission cycle.

Although USUV is considered a vector-borne pathogen, other routes of infection through shedding, such as the oropharyngeal route, have been suggested^19^. Non-vector transmission of USUV has been evaluated in experimentally infected canaries, transferring sputum from an infected canary to a naïve one, highlighting the potential relevance of the intranasal route of infection in blackbirds^19^. The present experiment shows the presence of a high load of USUV RNA in the pharyngeal and the cloacal swabs, progressively increasing from 1 d.p.i. These findings suggest oral and cloacal routes of shedding in blackbirds as potential transmission routes, that need, however, to be proven by the detection of infectious replicating virus.

Feathers have been proposed as a possible source of virus shedding following the detection of USUV antigen in the feather shaft of skin sections^9^. The potential role of feather sampling as a minimally invasive tool for USUV surveillance has been investigated by testing feathers using RT-qPCR^23^, which showed variable amounts of viral loads in different types of feathers. Contrary to what was reported in the literature, in this study USUV genome was detected in very low levels, only in the breast feathers of Afr3-infected blackbirds and not in Eu3-infected blackbirds at any sampling time. The reason behind the low detection of USUV in our experiment may stem from the anatomical region where the feathers were sampled. A previous study compared the amount of USUV RNA detected in the shaft of feathers collected from breast, wings and tail, and showed that viral RNA load obtained from chest feathers was lower compared to wing and tail feathers^23^. In the present study, breast feathers were chosen as clinical samples, instead of wing and tail feathers, to limit the stress of sampling on the blackbirds. The limited amount of USUV RNA retrieved on this type of feathers, even at the peak of viremia, might be due to the lower amount of blood present in the shaft of this type of feathers compared to the ones from the wing and tail, and confirms lower suitability of this type of sample for surveillance purposes.

We show that activity levels of blackbirds decrease steeply following an experimental USUV infection, while such a decrease in activity was lower in mock-infected blackbirds. While results on captive-bred blackbirds held in small cages are hard to extrapolate to activity patterns of wild birds in a more natural setting, these are in line with observations of lethargic, naturally USUV-infected blackbirds in the wild. A previous experimental field study on wild blackbirds showed that mimicking a bacterial infection (by injection of lipopolysaccharide) results in a strong reduction in activity levels of wild blackbirds within 24 h after infection, and a prolonged, more subtle effect on longer timescales^24^. This may indicate that the effects of infection on activity levels we observed in our study may not only result from neuro-invasion by the virus, but could also arise due to the energetic costs of mounting an immune response. Interestingly, the reduced activity we observed following USUV infection, could potentially result in a reduction in vector-avoidance behavior. If this is the case, USUV transmission rates may be enhanced by the inactivity of infected hosts. Future studies should focus on the effect of USUV infection on vector-avoidance behavior and the effect on virus transmission.

Based on the observation of the present study, blackbirds could be suitable models to investigate acute disease associated with USUV infection in a relevant host species. However, the limited commercial availability of blackbirds reduces the suitability of this species for experimental purposes. The detection of anti-USUV antibodies^23^ and pathological changes associated with a chronic stage of the disease (such as lymphoplasmacytic inflammatory infiltrate)^9^ in naturally infected blackbirds suggests that wild blackbirds survive longer after infection compared to the experimentally infected blackbirds of the present study. To model a later stage of USUV infection, lowering the dose of inoculation might allow for the progression of the disease to a chronic stage. Alternatively, other susceptible avian species might represent a suitable model for chronic infection in avian species, allowing a better understanding of chronic stage pathology, pathogenesis and immune response against the USUV.

In conclusion, our study shows that experimental USUV infection of blackbirds, through the subcutaneous route, leads to the development of severe disease with the presence of virus. The high levels of viremia suggest the relevance of blackbirds as a source for maintaining a transmission cycle between mosquitoes and birds, while the pharyngeal and cloacal shedding of the virus indicates a potential non-vectorial route of transmission between birds. However, the limited lifespan of the blackbirds following inoculation might reduce the probability of getting in contact with mosquitoes or other blackbirds, potentially limiting the chance of transmission. The acute development of the disease, virus titers in CNS, liver, spleen, heart and lung, as well as the nature of the histopathological lesions, suggest a possible early involvement of the peripheral organs and secondary spread to the CNS. The discrepancies related to neurological signs and histopathological changes of an acute disease not aligning with the findings of natural infection might indicate a more delayed course of the natural infection that needs to be further investigated.

## Methods

### Virus preparation

USUV Afr3 (isolated in 2016 from *Turdus merula*, GenBank accession MH891847.1) and USUV Eu3 (isolated in 2017 from *Turdus merula*, GenBank accession MN122189.1) were grown and passaged three times on Vero cells (African green monkey kidney epithelial cells, ATCC CCL-81). Cells were inoculated at a multiplicity of infection (MOI) of 0.01 and incubated at 37 °C for 5–6 days in Dulbecco’s modified Eagle’s medium (DMEM; Lonza) with 2% FBS (Sigma-Aldrich), 100 U/ml penicillin, 100 μg/ml streptomycin (Lonza), 1% sodium bicarbonate (Lonza) and 2 mM L-glutamine (Lonza). Supernatant was harvested, spun down at 4000×*g* for 10 min (mins), and subsequently aliquoted and stored at −80 °C. Stock titres were determined via titration on Vero cells. Both virus stocks were used at passage 3. For preparation of the blackbird inocula, viruses were diluted in sterile PBS to achieve end titres of 1 × 10^6^ TCID_50_/ml. Inocula were back-titrated to confirm inoculation titres of 5.62 × 10^4^ TCID_50_/100 µl for USUV Afr3 and 1 × 10^4^ TCID_50_/100 µl for USUV Eu3.

### Birds and ethical approval

Adult, male (*n* = 4) and female (*n* = 5) Eurasian blackbirds (*T. merula*) (*n* = 9) were obtained from amateur breeders in Belgium and the Netherlands and transferred to the licensed laboratory of the Erasmus Medical Center in Rotterdam (the Netherlands) with an approved OLAW Assurance # A5051-01. The blackbirds were singularly housed in cages (61 cm × 40 cm × 40cm) and randomly allocated in three separate isolators representing the Afr3-infected group (*n* = 3), the Eu3-infected group (*n* = 3) and the control group (*n* = 2). One blackbird (*n* = 1), that died prior to inoculation due to an unknown cause, was sampled and used as a negative control for tissue analysis. Commercial feed for fruit- and insect-eating birds (Versele-laga Nutribird Remline), fresh fruit, mealworms and fresh water were provided *ad libitum*. All animal procedures were conducted in compliance with the legislation for the protection of animals used for scientific purposes (2014, implementing EU Directive 2010/63) and other relevant regulations. The experiment was performed under a project license from the Dutch competent authority, and the study protocol (2200015) was approved by the institutional Animal Welfare Body.

### Challenge and sample collection

Upon arrival at the facility, all blackbirds were weighed and sampled as follows: 200 µl of blood were collected through venipuncture of the brachial wing vein into two 100 µl microvettes, containing respectively EDTA and clotting activator. Pharyngeal and cloacal swabbing were performed, and breast feathers were collected. To confirm negative status prior to infection of the blackbirds, serum samples from each blackbird were tested^25,26^ for the presence of neutralizing antibodies against USUV Afr3 (*T. merula* NL isolate, 2016) and WNV lineage 2 (B956, NCPV Porton Down #638, 2010) by a focus reduction neutralizing test (FRNT) as previously described with some modifications^25,26^.

**Fig. 7:**
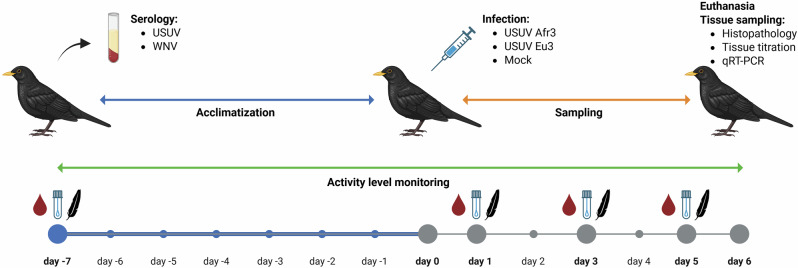
Experimental design. Eight Eurasian blackbirds were sampled on day -7 (blood, serum, pharynx and cloacal swabs and breast feathers); after sampling, the blackbirds were singularly housed in cages in isolators and left to acclimatize for 7 days and activity and health status were monitored. On day 0, blackbirds were either inoculated with USUV Afr3 (*n* = 3), Eu3 (*n* = 3), or sterile PBS (*n* = 2). On 1, 3 and 5 days post inoculation (d.p.i.) blackbirds were sampled (blood, serum, pharynx and cloacal swabs and breast feathers). At the humane endpoint, blackbirds were euthanized, necropsied, and tissue samples were collected for histopathology, tissue titration and RT-qPCR. Created in BioRender, agreement licence number OO28BUTARJ.

After 7 days of acclimatization in the isolators, all blackbirds were subcutaneously inoculated in the skin web of the right leg; one group of blackbirds (*n* = 3) was challenged with 1 × 10^4^ TCID50/100 µl of USUV Afr3, one group (*n* = 3) with 5 × 10^4^ TCID50/100 µl of USUV Eu3, and one group (*n* = 2) was mock-inoculated with 100 µl of sterile PBS (Fig. 7). To test for viremia and viral shedding through RT-qPCR, every other day starting from 1 day post infection (d.p.i.), 100 µl of blood was collected in EDTA as described above, pharyngeal and cloacal swabs were taken and breast feathers were collected (Fig. 1). When humane endpoints were reached, the animals were anesthetized by 5% isoflurane inhalation and euthanized through exsanguination via intracardiac puncture. Humane endpoints were established as follows: (1) Loss of more than 25% of body weight compared to the start of the experiment; (2) onset of neurological symptoms such as head shaking, head and neck tremors, loss of balance, circling, wing and leg paralysis, seizures; (3) significant deviation of behavior and movement pattern from routine. Necropsy was performed to assess the macroscopic lesions and to collect tissue samples (pallium, spinal cord, liver, spleen, heart, kidney, lung, proventriculus and gizzard, intestine, eye, and skin) for histopathology, immunohistochemistry, tissue titration, and RT-qPCR.

### Activity patterns of blackbirds in the infection trials

Activity levels of blackbirds were quantified using trail cameras (Reconyx HC500 Hyperfire) placed on tripods in front of each of the isolators. Each camera was set to take one image every minute throughout the experiment, using an infrared flash (invisible to the blackbirds) to illuminate the cages during the night. From these images, the position of each blackbird within its cage was extracted using a custom Python script (Python v. 3.10^20^ and 3.10)^27^. All moved distances within each hour were summed to obtain hourly individual activity measurements (back-transformed to meters using the dimensions of the cage on the image as a reference). USUV infection influence on activity levels of blackbirds was assessed using these hourly activity measurements. If a USUV infection results in decreased activity levels in blackbirds, a decline in activity levels was expected over time since infection, while such a decrease should be less pronounced in mock-infected blackbirds. To test this hypothesis, a general additive mixed effects model (GAMM) was run using the mgcv package in R v.4.2.2^21^ (detailed methods in Supplementary materials).

### Histopathology and immunohistochemistry

Tissues collected during necropsy were fixed in 4% buffered formaldehyde. After processing of the tissues and embedding them in paraffin, 3–5-μm-thick sections were cut and subsequently stained in hematoxylin and eosin (H&E) for histopathological investigation under light microscopy. Tissues were assessed for histological lesions, and the main target organs were scored (liver, heart, spleen, pallium, and heart) based on the scoring system as described previously^9^.

For the detection of USUV antigen, tissue sections of liver, heart, spleen, pallium, heart, kidney, lung, proventriculus and gizzard, small and large intestine, eye, and skin were stained with a polyclonal rabbit serum (U433, rabbit vaccinated with inactivated USUV Germany) generously provided by Friedrich-Loeffler-Institut, Insel Riems, Germany^28^ as previously described^9^. The presence of USUV antigen on IHC-stained tissues was scored with a two-tier scoring system as described previously^9^.

### Virus titration from tissue samples

Tissue samples collected during necropsy were homogenized in 10% w/v ratio in virus transport medium (500 ml MEM eagle medium with hank salts, 2.5 g lactalbumin enzymatic hydrolysate, 12 ml pen strep 10,000 U/ml, 6 ml polymyxin B sulphate, 3 ml nystatin, 3 ml gentamycin, 60 ml glycerol, 12.5 ml HEPES) in MagNA lyser green beads tube using a MagNA lyser (Roche diagnostics). Tenfold serial dilutions of homogenates were inoculated onto a semiconfluent monolayer of Vero cells seeded onto a 96-well plate (2.3 × 10^4^ cells/well) 24 h prior. Cytopathic effect (CPE) was used as a titer readout and determined at 6 d.p.i. Virus titres were calculated as the 50% tissue culture infective dose (TCID_50_) using the Spearman–Kärber method^29^. The preparation of a 10% solution and the initial 1:10 dilution of supernatant resulted in a detection limit of 316 TCID_50_/ml.

### RNA extraction and RT-qPCR

RNA extraction was performed using the RNeasy Plus mini kit (Qiagen). Feathers, pallium and lung were homogenized as described above. About 10 µL of blood or 60 µL of 10% w/v tissue homogenate was placed in 350 µL RLT buffer (Qiagen), and cloaca swabs were placed in 500 µL RLT buffer. For RNA extraction, the RNeasy Plus mini kit protocol was used. Real-time RT-qPCR was done using an iTaq universal probes one-step kit. The reaction mix contained 10 µM forward primer (5’-CAAAGCTGGACAGACATCCCTTAC-3’), 10 µM reverse primer (5’-CGTAGATGTTTTCAGCCCACGT-3’), 10 µM probe (5’-AAGACATATGGTGTGGAAGCCTGATAGGCA-3’), 1x iTaq universal probes reaction mix, 1x iScript reverse transcriptase, and 2 µL of RNA^30,31^. A thermal cycling protocol was performed in a CFX connect PCR machine (Bio-Rad) as follows: reverse transcription at 50 °C for 20 min, polymerase inactivation at 95 °C for 5 min, denaturation at 95 °C for 15 s, and annealing/extension at 60 °C for 35 s. Denaturation, annealing/extension steps were repeated for 45 cycles.

### Focus reduction neutralizing test (FRNT)

Sera were heat-inactivated for 30 min at 56 °C. The sera were twofold serially diluted, starting with 1:10. Thereafter, a virus suspension (800 focus-forming units (FFU) based on 24-h titrations) was added to a final concentration per well of 400 FFU and incubated for 1 h at 37 °C. Virus and serum mix was added to a confluent monolayer of Vero cells and incubated for 24 h at 37 °C. After 24 h, cells were fixed with 4% paraformaldehyde (PFA), permeabilized with 0.5% TritonTM X-100 – PBS (0.5% v/v, Merck, T8787-50ML) and blocked with 5% Blocker Blotto in TBS. Cells were stained with polyclonal mouse anti-USUV NS1 antibody (1:4000, MyBioscource, MBS569354_1mg) or anti-WNV NS1 antibody (1:4000, IC12) (The Native Antigen Company, MAB12160–100) diluted in Blocker blotto, followed by secondary antibody staining with goat anti-mouse IgG(H + L) cross-adsorbed horseradish peroxidase (HRP) (1:6000, Invitrogen, A16072). After and in between primary and secondary antibody staining, cells were incubated for 1 h (37 °C, 5% CO_2_) and washed with PBS. Thereafter TrueBlue peroxidase substrate (KPL TrueBlue, 5510–0030, Seracare) was added to the wells and incubated in the dark at room temperature for 5–10 min. Plates were washed with PBS, air-dried and scanned by the CTL Immunospot scanner (S6 Ultimate-V Analyzer, CTL Analyzers LCC). FRNT titers were calculated based on a 90% reduction in infected cell counts. A reciprocal titer of ≥1:160 for USUV, a ≥1:80 for WNV and a ≥fourfold difference between WNV and USUV FRNT titers was considered as positive.

### Statistical analysis

Statistical analysis comparing the average weight loss, viremia, pharyngeal shedding and cloacal shedding between the three groups of birds was performed on GraphPad Prism 10.1.1 by mixed effect analysis (alpha = 0.05). Statistical analysis comparing the average amount of replicating virus expressed in TCID_50_/g detected by tissue titration and the average amount of viral RNA expressed in TCID_50_ equivalents/g detected by RT-qPCR was performed using a two-way ANOVA test (alpha = 0.05). The probability of survival comparing the three groups of blackbirds was analysed using the log-rank (Mantel–Cox) test. To test whether a USUV infection results in decreased activity levels in blackbirds compared to control blackbirds, we ran a general additive mixed effects model (GAMM) using the mgcv package in R v.4.2.2^32^.

## Supplementary information

Arrive checklist

Supplementary Materials

## Data Availability

The datasets used and/or analysed during the current study are available from the corresponding author upon reasonable request.

## Code availability

N/A.
