# Supplementary material for: Experimental Usutu virus infection in Eurasian blackbirds (Turdus merula)
Source: Npj Viruses. 2025 Jun 20;3:51. doi: 10.1038/s44298-025-00133-w (PMC12181337; doi:10.1038/s44298-025-00133-w)
Supplement: Supplementary file 1 — Arrive checklist [file 44298_2025_133_MOESM1_ESM.pdf]

Supplementary materials

Model selection

Family: gaussian
Link function: identity

Formula:
sqrt(sum_moved_distance) ~ Treatment*days.pi.scaled*stage +
s(hours.day, bs = "bs", by = Individual)

Parametric Terms:

	df	F	p-value
Treatment	1	2.055	0.15183
days.pi.scaled	1	29.135	7.60e-08
stage	1	8.474	0.00364
Treatment:days.pi.scaled	1	35.789	2.62e-09
Treatment:stage	1	33.652	7.71e-09
days.pi.scaled:stage	1	15.284	9.58e-05
Treatment:days.pi.scaled:stage	1	8.848	0.00297

Approximate significance of smooth terms:

	edf	Ref.df	F	p-value
s(hours.day):Individual1	5.547	6.525	21.85	<2e-16
s(hours.day):Individual2	6.893	7.772	32.41	<2e-16
s(hours.day):Individual3	8.043	8.684	29.91	<2e-16
s(hours.day):Individual4	5.655	6.629	31.25	<2e-16
s(hours.day):Individual5	7.319	8.142	22.35	<2e-16
s(hours.day):Individual6	6.183	7.129	15.58	<2e-16

Minimal adequate model summary

Family: gaussian, Link function: identity

Formula:
sqrt(sum_moved_distance) ~ Treatment*days.pi.scaled*stage +
s(hours.day, bs = "bs", by = Individual)

Parametric coefficients:

	Estimate	Std. Error	t value	Pr(>|t|)	
(Intercept)	0.74981	0.01522	49.254	< 2e-16	***
Treatment(USUV)	0.02936	0.02048	1.434	0.15183	
days.pi.scaled	-0.07809	0.01447	-5.398	7.60e-08	***
stage(pre)	0.08517	0.02926	2.911	0.00364	**
Treatment(USUV):days.pi.scaled	-0.12239	0.02046	-5.982	2.62e-09	***
Treatment(USUV):stage(pre)	0.22168	0.03821	5.801	7.71e-09	***
days.pi.scaled:stage(pre)	0.11331	0.02898	3.909	9.58e-05	***
Treatment(USUV):days.pi.scaled:stage(pre)	0.11395	0.03831	2.975	0.00297	**

Signif. codes: 0 ‘***’ 0.001 ‘**’ 0.01 ‘*’ 0.05 ‘.’ 0.1 ‘ ’ 1

Approximate significance of smooth terms:

	edf	Ref.df	F	p-value	
s(hours.day):Individual1	5.547	6.525	21.85	<2e-16	***
s(hours.day):Individual2	6.893	7.772	32.41	<2e-16	***
s(hours.day):Individual3	8.043	8.684	29.91	<2e-16	***
s(hours.day):Individual4	5.655	6.629	31.25	<2e-16	***
s(hours.day):Individual5	7.319	8.142	22.35	<2e-16	***
s(hours.day):Individual6	6.183	7.129	15.58	<2e-16	***

Signif. codes: 0 ‘***’ 0.001 ‘**’ 0.01 ‘*’ 0.05 ‘.’ 0.1 ‘ ’ 1

R-sq.(adj) = 0.441   Deviance explained = 45.4%
-REML = 924.25 Scale est. = 0.13911   n = 1938

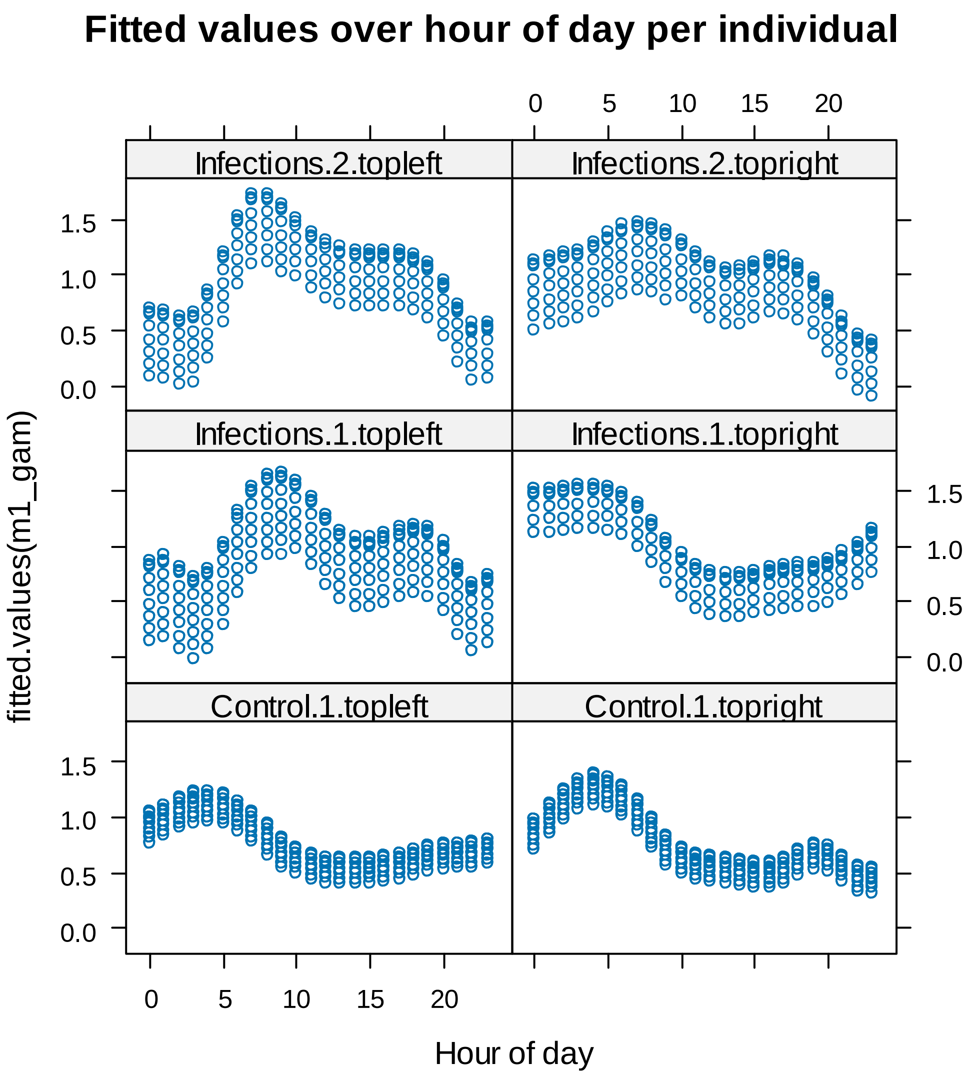

Supplementary figure 1. **Fitted values over hour of day per individuals.** Smoothers for each individual's activity over 'hour of day' (fitted values).

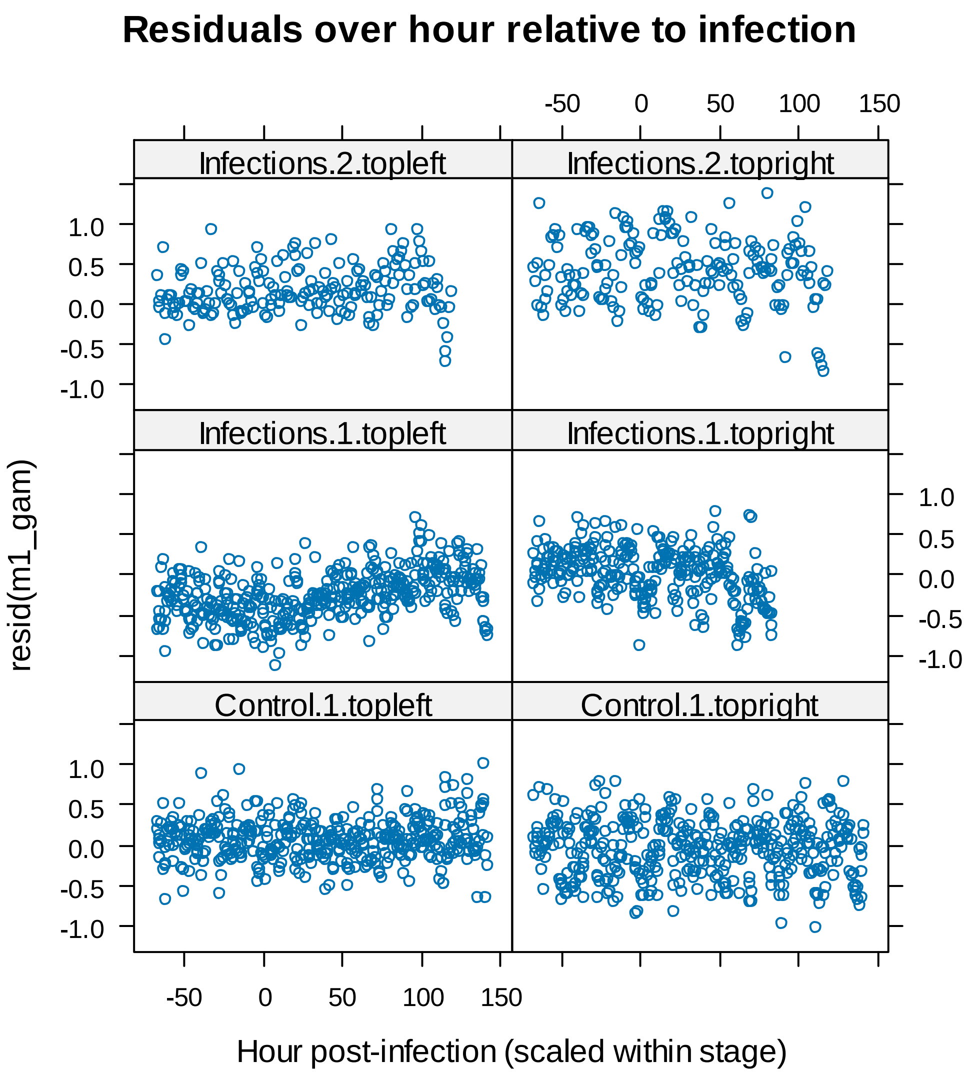

Supplementary figure 2. **Residuals of the minimal adequate model fit over hours post-infection**. The model was fit to days.pi (scaled within pre vs post-infection stage), and this figure is only included to show the absence of any patterns in the residuals when including the smooth term.

Supplementary table 1. results of RT-qPCR analysis on blood collected at 0;1;3 and 5 days post infection (d.p.i.), expressed in Ct value. A value of 0,00 have been applied to Ct values above 45 as conventional cut-off value to consider a tested sample negative.

	**Afr3**			**Eur3**			**Controls**	
**d.p.i**	**BB1**	**BB2**	**BB4**	**BB3**	**BB5**	**BB6**	**BB8**	**BB9**
**0**	0,00	0,00	0,00	0,00	0,00	0,00	0,00	0,00
**1**	27,58	19,31	19,81	21,75	24,85	21,73	0,00	0,00
**3**	18,89	20,47	12,13	22,15	23,20	16,25	0,00	0,00

**5**	24,20	26,93		18,28	24,73	21,60	0,00	0,00

Supplementary table 2. results of RT-qPCR analysis on pharyngeal swabs collected at 0;1;3 and 5 days post infection (d.p.i.), expressed in Ct value. A value of 0,00 have been applied to Ct values above 45 as conventional cut-off value to consider a tested sample negative.

	**Afr3**			**Eur3**			**Controls**	
**d.p.i**	**BB1**	**BB2**	**BB4**	**BB3**	**BB5**	**BB6**	**BB8**	**BB9**
**0**	0,00	0,00	0,00	0,00	0,00	0,00	0,00	0,00
**1**	37,99	34,92	0,00	39,88	36,75	36,67	0,00	0,00
**3**	31,28	25,06	22,89	26,30	25,13	27,82	0,00	0,00
**5**	23,02	21,49				19,51	0,00	0,00

Supplementary table 3. results of RT-qPCR analysis on cloacal swabs collected at 0;1;3 and 5 days post infection (d.p.i.), expressed in Ct value. A value of 0,00 have been applied to Ct values above 45 as conventional cut-off value to consider a tested sample negative.

	**Afr3**			**Eur3**			**Controls**	
	**BB1**	**BB2**	**BB4**	**BB3**	**BB5**	**BB6**	**BB8**	**BB9**
**0**	0,00	0,00	0,00	0,00	0,00	0,00	0,00	0,00
**1**	0,00	37,01	36,19	34,62	41,14	36,15	0,00	0,00
**3**	30,24	25,89	31,10	24,88	0,00	23,70	0,00	0,00
**5**	23,02	27,52				20,41	0,00	0,00

Supplementary table 4. results of RT-qPCR analysis on breast feathers collected at 0;1;3 and 5 days post infection (d.p.i.), expressed in Ct value. A value of 0,00 have been applied to Ct values above 45 as conventional cut-off value to consider a tested sample negative.

	**Afr3**			**Eur3**			**Controls**	
	**BB1**	**BB2**	**BB4**	**BB3**	**BB5**	**BB6**	**BB8**	**BB9**
**0**	0,00	0,00	0,00	0,00	0,00	0,00	0,00	0,00
**1**	0,00	37,88	31,30	0,00	38,74	38,08	0,00	0,00
**3**	0,00	35,56	37,12	34,65	0,00	0,00	0,00	0,00
**5**	0,00	35,02				35,58	0,00	0,00

Supplementary table 5. results of virus titration on tissues samples collected during necropsy, expressed in TCID50.

	**Afr3**			**Eur3**			**Control**
	**BB1**	**BB2**	**BB4**	**BB3**	**BB5**	**BB6**	**BB7**
**Pallium**	$1,47x10^{7}$	$3.16x10^{5}$	$6.81x10^{6}$	$3.16x10^{3}$	$3.16x10^{4}$	$3.16x10^{5}$	$3.16x10^{1}$

**Cerebellum**	$3.16x10^{6}$	$3.16x10^{7}$	$6.81x10^{6}$	$6.81x10^{6}$	$3.16x10^{5}$	$3.16x10^{6}$	$3.16x10^{1}$
**Spinal cord**	$3.16x10^{5}$	n.a.	n.a.	n.a.	$1,47x10^{6}$	n.a.	$3.16x10^{1}$
**Liver**	$3.16x10^{2}$	$6.81x10^{2}$	$1,47x10^{7}$	$1,47x10^{3}$	$1,47x10^{4}$	$1,47x10^{5}$	$3.16x10^{1}$
**Spleen**	$6.81x10^{2}$	$3.16x10^{4}$	$1,47x10^{5}$	$1,47x10^{5}$	$3.16x10^{5}$	$6.81x10^{4}$	$3.16x10^{1}$
**Heart**	$6.81x10^{5}$	$3.16x10^{4}$	$3.16x10^{6}$	$1,47x10^{5}$	n.a.	$3.16x10^{5}$	$3.16x10^{1}$
**Kidney**	$1,47x10^{5}$	$6.81x10^{4}$	$3.16x10^{7}$	$6.81x10^{4}$	$3.16x10^{5}$	$3.16x10^{6}$	$3.16x10^{1}$
**Lung**	$3.16x10^{7}$	$6.81x10^{7}$	$6.81x10^{9}$	$6.81x10^{6}$	$1,47x10^{5}$	$3.16x10^{8}$	$3.16x10^{1}$
**Proventriculus and Gizzard**	$3.16x10^{5}$	$6.81x10^{3}$	$6.81x10^{3}$	$6.81x10^{2}$	$3.16x10^{2}$	$6.81x10^{5}$	$3.16x10^{1}$
**Intestine**	$3.16x10^{4}$	$3.16x10^{4}$	$3.16x10^{4}$	$3.16x10^{3}$	$3.16x10^{2}$	$3.16x10^{3}$	$3.16x10^{1}$
**Eye**	$1,47x10^{5}$	$6.81x10^{5}$	$3.16x10^{7}$	$3.16x10^{5}$	$1,47x10^{5}$	$3.16x10^{6}$	$3.16x10^{1}$
**Skin (inoc.)**	$3.16x10^{4}$	$1,47x10^{3}$	$3.16x10^{8}$	$6.81x10^{6}$	$1,47x10^{6}$	$6.81x10^{5}$	$3.16x10^{1}$
**Skin**	n.a.	$3.16x10^{3}$	$3.16x10^{2}$	$3.16x10^{5}$	$3.16x10^{2}$	$3.16x10^{2}$	$3.16x10^{1}$

Supplementary table 6. results of RT-qPCR analysis on tissues samples collected during necropsy, expressed in Ct values.

	**Afr3**			**Eur3**			**Control**
	**BB1**	**BB2**	**BB4**	**BB3**	**BB5**	**BB6**	**BB7**
**Pallium**	18,71	19,75	17,18	19,41	17,91	17,77	0,00
**Lung**	20,87	18,79	13,50	0,00	19,37	15,93	0,00
